# miR-200b is involved in intestinal fibrosis of Crohn’s disease

**DOI:** 10.3892/ijmm.2012.894

**Published:** 2012-01-23

**Authors:** YINGWEI CHEN, WENSONG GE, LEIMING XU, CHUNYING QU, MINGJIE ZHU, WENZHU ZHANG, YONGTAO XIAO

**Affiliations:** 1 Department of Gastroenterology, Shanghai Jiao Tong University, School of Medicine, Xin Hua Hospital, Shanghai; 2 Shanghai Institute of Pediatric Research, Shanghai; 3 Shanghai Key Laboratory of Pediatric Gastroenterology and Nutrition, Shanghai; 4 Department of Pathology, Shanghai Jiao Tong University, School of Medicine, Xin Hua Hospital, Shanghai, P.R. China

**Keywords:** Crohn’s disease, diagnosis, microRNA, epithelial-mesenchymal transition, fibrosis

## Abstract

Intestinal fibrosis is one of the major serious complications of Crohn’s disease (CD). However, there are no effective antifibrotic drugs to treat intestinal fibrosis in CD. Therefore, it is important to understand the pathogenesis of fibrosis in CD. It has been reported that members of the miR-200 family are essential in the regulation of renal fibrogenesis. In this study, we analyzed the function of miR-200a and miR-200b in intestinal fibrosis, which was induced by transforming growth factor β1 (TGF-β1) *in vitro*. Furthermore, we detected the expression of miR-200a and miR-200b in CD specimens, which were divided into groups of fibrosis and no-fibrosis. The results of this study showed that administration of miR-200b could partially protect intestinal epithelial cells from fibrogenesis *in vitro*. Furthermore, we found that miR-200b was overexpressed in the serum of the fibrosis group. The results suggest that miR-200b has potential value for diagnostic and therapeutic applications for CD patients with fibrosis complications.

## Introduction

Fibrosis is one of major complications in Crohn’s disease (CD), which results from chronic inflammation. Severe fibrosis could result in critical narrowing of the lumen stenosis, or stricture, commonly leading to obstruction that requires surgical or mechanical intervention (1–3). At present, there are no approved or effective medical therapies aimed specifically at fibrosis or stricture in CD. Anti-inflammatory therapies do not prevent fibrosis nor do they reverse established strictures, which may present years after remission of active inflammation. Although this study is still limited, it can expand our understanding of cellular and molecular mechanisms leading to fibrosis in CD.

microRNAs (miRNAs) are noncoding RNAs that negatively regulate target genes expression at the post-transcriptional level. There is growing evidence to suggest that members of miR-200 family are implicated in diverse biological and pathological processes. Recent ground-breaking studies have established functional associations between miR-200 and key effectors of the epithelial-to-mesenchymal-transition (EMT) occurring in the context of carcinogenesis and embryonic development. Gregory *et al* (4) reported that miR-200 and miR-205 could inhibit the zinc finger E-box-binding homeobox 1 (ZEB1) and ZEB2, ZEB1 (TCF8/EF1) and ZEB2 [SMAD-interacting protein 1 (SIP1)/ZFXH1B] and thus maintain the epithelial cell phenotype (4). Oba *et al* (5) indicated that a miR-200b precursor could ameliorate renal tubulointerstitial fibrosis. The purpose of this study was to investigate the role of miR-200a and miR-200b in the pathogenesis of intestinal fibrosis. Initial experiments demonstrated that introduction of miR-200b in intestinal epithelial cells could partially oppose fibrosis, which was induced by transforming growth factor β1 (TGF-β1). Subsequent data showed the aberrant expression of miR-200b in serum is a potential diagnostic marker for CD patients with fibrosis complications.

## Materials and methods

### TGF-β1-induced fibrosis in vitro

DLD-1 was a colorectal adenocarcinoma epithelial cell line, which was cultured in DMEM (Gibco) containing 10% fetal bovine serum (FBS). DLD-1 cells were stimulated with 10 ng/ml (TGF-β1, Sigma) for 24, 48 and 72 h. The total-RNA and protein were harvested at the above-indicated times. To investigate the effect of miRNAs on fibrosis, DLD-1 cells were transfected with 50 pM miR-200a or miR-200b for 24 h using Lipofectamine RNAiMAX (Invitrogen), then stimulated with 10 ng/ml TGF-β1. After 24 h, the cells were harvested by extracting total-RNA and protein.

### miRNA assays

Total-RNA was extracted from serum, tissues of patients and DLD-1 cells by using the mirVana PARIS and miRVana miRNA isolation kit (Ambion). TaqMan miRNA assay (Applied Biosystems) was used to quantify the relative expression level of miR-200a (assay ID. 000502), miR-200b (assay ID. 002251), and U6 (assay ID. 001093) was used as an internal control. cDNA was synthesized using the TaqMan miRNA Reverse Transcription kit (Applied Biosystems). The reaction was performed for 30 min at 16°C, 30 min at 42°C, and 5 min at 85°C. The LightCycler^®^ 480 Real-Time PCR System (Roche) was used to detect miRNA expression. All reactions were run in triplicate.

### Real-time PCR

The total-RNA was extracted from DLD-1 cells with TRIzol (Invitrogen) according to the protocol of manufacture. Real-time PCR was performed to measure the expression of vimentin, fibronectin, E-cadherin, fibronectin. Real-time PCR was performed with the following PCR primers: *GAPDH*, forward, 5′-GAAGGTGAAGGTCGGAGTC-3′ and reverse, 5′-GAAGATGGTGATGGGATTTC-3′; *CDH1*, forward, 5′-GGAGGAGAGCGGTGGTCAAA-3′ and reverse, 5′-TGTG CAGCTGGCTCAAGTCAA-3′; *vimentin*, forward, 5′-CCTC CTACCGCAGGATGTT-3′ and reverse, 5′-CTGCCCAGG CTGTAGGTG-3′; *α-SMA*, forward, 5′-CCGACCGAATGCA GAAGGA-3′ and reverse, 5′-ACAGAGTATTTGCGCTCCG AA-3′; *fibronectin*, forward, 5′AGACCATACCTGCCGAATG TAG-3′ and reverse, 5′-GAGAGCTTCCTGTCCTGTAGAG-3′; SYBR-Green Universal Master Mix kit (ABI) and High Capacity cDNA Reverse Transcription kit (ABI) were employed to detect the levels of these genes. All reactions were repeated four times and GAPDH was used to normalize the target genes.

### Western blotting

Protein of 20 μg/well was separated on 4–12% SDS-polyacrylamide gels and transferred onto nitrocellulose membranes (Invitrogen) using a dry blotting system (Invitrogen). After blocking in 1X TBST, 5% nonfat dry milk, 0.2% Tween-20 at room temperature for 30 min. The membranes were incubated with the primary antibodies in blocking buffer (1X TBST, 3% nonfat dry milk, 0.2% Tween-20) overnight at 4°C. Antibodies were used at a dilution of 1:1,000. The membranes were washed three times for 30 min with 1X TBST and then incubated with secondary antibodies at a final dilution of 1:2,000. After final washes with 1X TBS, 0.2% Tween-20, the signals were detected using ECL chemiluminescence reagents (Pierce). Antibodies of N-cadherin, E-cadherin, vimentin and actin α2 smooth muscle (α-SMA) were used in this assay. To confirm that the same amount of protein was investigated, the expression of GAPDH was investigated simultaneously.

### Specimen preparation

A total of 20 tissue specimens from CD patients (10 fibrosis samples and 10 no-fibrosis samples) were obtained from the Department of Pathology, Xinhua Hospital, Shanghai, China. The blood samples were donated from the above patients (Table I). All of the patients or their guardians provided written informed consent, and Faculty of Medicine’s Ethics Committee of Xinhua Hospital approved all aspects of this study.

### Immunohistochemistry

Immunohistochemical studies for the expression of N-cadherin, E-cadherin, vimentin, and α-SMA were performed on 20 CD samples by using an avidin-biotin peroxidase method with diaminobenzidine (DAB) chromogen. After antigen retrieval (microwave treatment of formalin-fixed, paraffin-embedded, 40 min at 240 W in citrate buffer, pH 6.0), the tissues were blocked with bovine serum albumin (BSA). As primary antibodies, mouse monoclonal and rabbit polyclonal antibodies (N-cadherin, Novus, dilution 1:200; E-cadherin, Cell Signal Technology, dilution 1:200; vimentin, Cell Signal Technology, 1:100; α-SMA, Novus, dilution 1:200) were used in this study. After incubation for 30 min at 37°C the slides were rinsed in phosphate-buffered saline (PBS) and incubated with the secondary antibody for 2 h at room temperature. Antibody binding was visualized with 0.05% DAB and 0.01% hydrogen peroxide. The material was rinsed in PBS and counterstained with hematoxylin.

### Statistical analysis

All data are presented as mean ± SD. When comparisons were made between two different groups, statistical significance was determined by the Student’s t-test using the StatView software program.

## Results

### Established TGF-β1-induces fibrosis in vitro

**Figure 1:**
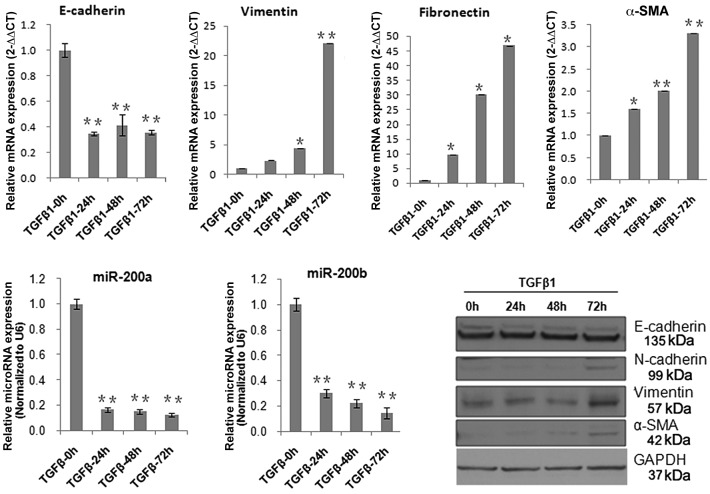
TGF-β1-induces fibrosis *in vitro* and inhibits the expression of miR-200a and miR-200b. After stimulating DLD-1 for 24, 48, 72 h with TGF-β1, the markers of fibrosis (vimentin, fibronectin, α-SMA) increased significantly at both the mRNA and protein levels, while the markers of EMT also increased. Meanwhile, the expression of miR-200a, miR-200b were downregulated by TGF-β1.

It is well accepted that TGF-β is a major inducer of fibrosis and has different isoforms. High levels of TGF-β1 have often been found in many fibrotic tissues, and have been implicated as a mediator of fibrosis in many diseases. Furthermore, TGF-β1 was firstly identified as an inducer of EMT in normal mammary epithelial cells and has since been shown to mediate EMT in a number of different epithelial cells, including renal proximal tubular and lens cells (6–9). Here, in order to clarify the effect of miR-200a and miR-200b on intestinal fibrosis, we initially established a TGF-β1-induced fibrotic model *in vitro* in a colorectal epithelial cell line (DLD-1). In this model, the DLD-1 cells were stimulated with TGF-β1 (10 ng/ml) for 24, 48 and 72 h. Real-time PCR and western blot analysis demonstrated that TGF-β1 mediated repression of E-cadherin, and induction of N-cadherin, α-SMA, fibronectin, and vimentin. E-cadherin was known to be involved in homophilic interactions between epithelial cells and was necessary for the formation of zonulae occludens. In the normal intestinal epithelial cells, E-cadherin staining was strong at the lateral cell membrane between cell contacting sites (10). However, E-cadherin expression was lost or was significantly reduced during the process of EMT. N-cadherin, which is usually expressed in mesenchymal cells and fibroblasts, has often been used to monitor the progress of EMT and fibrogenesis (11,12). Vimentin is a type III intermediate filament protein that is expressed in mesenchymal cells and fibroblasts (13,14). During the development of intestinal fibrosis in responding to chronic pressure or volume overload, fibroblasts could become activated to become myofibroblasts, which strongly express α-SMA and secrete abundant, disorganized collagen (15–17). The above data indicate that TGF-β1 not only induced fibrosis, but also the EMT process. At the same time, we found that the expression of miR-200a and miR-200b were significantly inhibited by TGF-β1 (Fig. 1).

### miR-200b ameliorates TGF-β1-induced fibrosis

**Figure 2:**
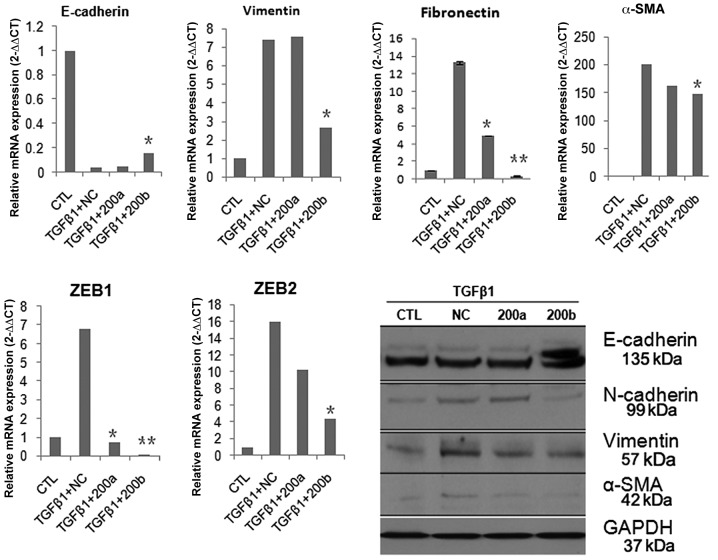
miR-200b protects from intestinal fibrosis *in vitro*. Real-time PCR and western blotting demonstrated that the markers of fibrosis or EMT were inhibited by miR-200b. Furthermore, ZEB1 and ZEB2 were downregualted by miR-200b. Thus, miR-200b may ameliorate fibrosis through regulation of ZEB1 and ZEB2.

We next investigated the role of miR-200a and miR-200b in intestinal fibrogenesis *in vitro*, with TGF-β1 stimulated DLD-1 cells for 24 h prior to introduction of miR-200a or miR-200b. miR-200b, but not miR-200a, increased the expression of E-cadherin and simultaneously reduced the expression of vimentin, fibronectin and N-cadherin (Fig. 2). It was thus suggested that miR-200b could ameliorate the fibrosis or EMT of intestinal epithelial cells *in vitro*. Recent studies have indicated that the transcriptional repressors ZEB1 (TCF8/EF1) and ZEB2 [SMAD-interacting protein 1 (SIP1)/ZFXH1B] are two direct targets of members of the miR-200 family (4). Here, real time PCR analysis showed that ZEB1 and ZEB2 were significantly inhibited by miR-200a or miR-200b (Fig. 2). These results support the notion that miR-200b could ameliorate intestinal fibrosis through downregulation of ZEB1, ZEB2.

### miR-200 is related to fibrogenesis of CD

**Figure 3:**
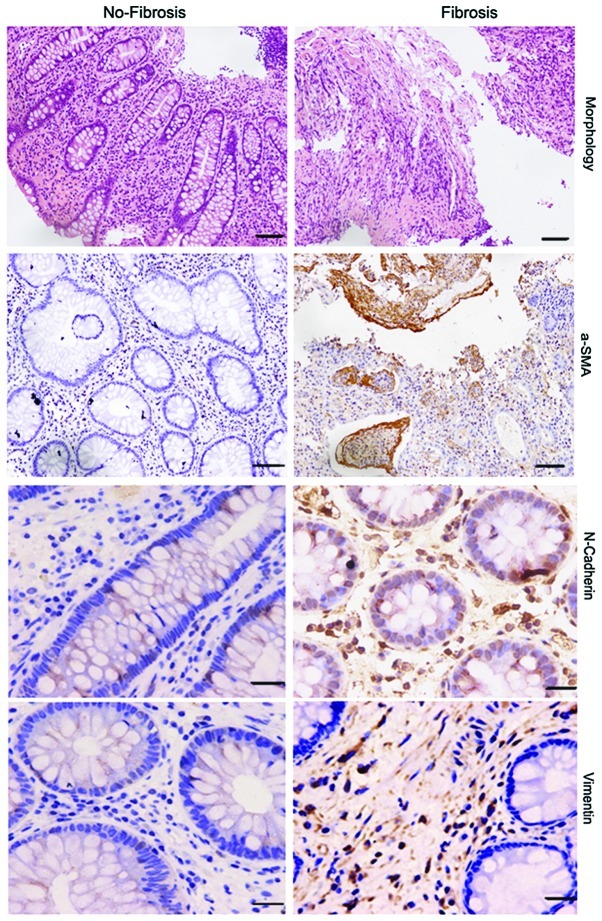
Evidence of fibrosis and EMT in the Crohn’s disease (CD) specimens. Immunohistochemistry was carried out to detect markers of fibrosis and EMT in CD specimens. Staining for α-SMA and vimentin (bar, ×400), suggesting the presence of fibrosis, was strong in some CD specimens. In parallel, the staining of N-cadherin was positive in fibrosis specimens when compared to the no-fibrosis ones. Changes in morphology were also observed (bar, ×200).

Intestinal fibrosis is a major complication of CD, but the precise mechanism is only partially understood. As a result, the exploration of specific therapies to halt fibrosis have been hindered. Here, we evaluated the possible contribution of the epithelial to mesenchymal transition (EMT) to intestinal fibrosis associated with CD. We detected the expression of specific markers of EMT and fibrosis in twenty CD specimens (10 fibrosis, and 10 no-fibrosis) by immunohistochemistry. Compared to the no-fibrosis specimens, we found that staining of N-cadherin, α-SMA and vimentin was very strong in fibrosis specimens (Fig. 3, Table I). These results prompted us to assess whether miR-200a or miR-200b are related to fibrogenesis of CD.

### Serum miR-200b is a potential diagnostic marker for CD with fibrosis complications

**Figure 4:**
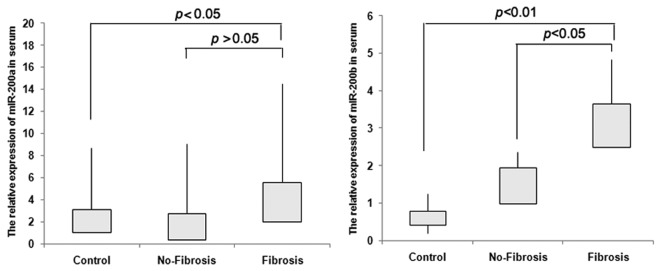
miR-200b levels in serum of CD fibrosis patients. miR-200b, but not miR-200a, significantly increased in both fibrosis and no-fibrosis patients’ serum when compared to serum of healthy subjects (n=10, 10, 16). However, it seemed that miR-200b was significantly elevated in the serum of CD fibrosis patients when compared to no-fibrosis ones.

In previous studies, Murakami *et al* (18) showed that overexpression of miR-200b could be connected to the progression of liver fibrosis. In order to investigate whether miR-200a or miR-200b could serve as diagnostic markers for CD fibrosis, we evaluated their expression in CD serum. We collected 10 fibrosis and 10 no-fibrosis blood samples and calculated the expression of miR-200a and miR-200b by TaqMan real-time PCR. Sixteen healthy blood samples were used as negative control. The results indicate that miR-200b increased significantly in serum of the fibrosis group when compared to that of the no-fibrosis group or from healthy individuals, (P<0.05, P<0.01). For miR-200a, we could not find a significant difference between the fibrosis and no-fibrosis groups (P>0.05) (Fig. 4).

## Discussion

Intestinal fibrosis is a common complication of CD that may require surgical intervention if it became seriously symptomatic. The traditional mechanisms underlying intestinal fibrosis are associated to the presence of chronic inflammation. However, it is also possible that novel mechanisms independent of persistent immune activation exist in the gut (18,19). At present, the development of a preventive and more effective management of intestinal fibrosis is hampered by the lack of a in-depth understanding of the basic pathophysiological factors involved in it. To provide novel mechanisms for intestinal fibrosis, we revealed the association between miR-200 and intestinal fibrosis. Initially, we analyzed the role of miR-200a and miR-200b in the intestinal fibrosis *in vitro*. The findings indicate that miR-200b could ameliorate intestinal fibrosis that was induced by TGF-β1. Several studies have shown that the members of the miR-200 family could target ZEB1 and ZEB2 (4) directly. Here, we indicated that ZEB1 and ZEB2 decreased significantly in the presence of miR-200a or miR-200b. It was suggested that miR-200b inhibited the process of intestinal fibrosis through repressing ZEB1 and ZEB2. However, more studies are needed to investigate the exact mechanisms involved (Figs. 1 and 2).

To provide further evidence for intestinal fibrosis, we revealed the association between miR-200 expression and CD fibrosis. We divided CD samples into two groups: fibrosis and no-fibrosis. Then, we evaluated the expression of miR-200a and miR-200b in the serum of CD samples. The results showed that miR-200b was significantly elevated in the group of CD fibrosis compared to the no-fibrosis group or the healthy controls, but surprisingly, miR-200a did not increase (Fig. 4). miRNAs are usually described as highly tissue-specific biomarkers with potential clinical applicability for defining the cancer origin of metastases. Recent studies suggested that circulating miRNA has been a valuable biomarker for several diseases, including different types of cancer (20–39). In this study, it was shown that expression of miR-200b increased significantly in the serum of the CD fibrosis group. This led us to hypothesize that serum miR-200b could be a candidate CD fibrosis diagnostic biomarker.

**Figure 5:**
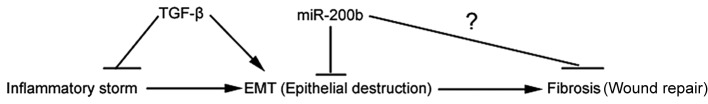
Schematic representation of the hypothesis that miR-200b is involved in CD fibrosis. Intestinal epithelial cells transition into mesenchymal cells (EMT) in the presence of stimulating inflammatory cytokines. TGF-β1 increases to oppose the process of inflammation, leading to fibrosis or EMT. miR-200 may ameliorate intestinal fibrosis possibly through inhibiting the EMT. However, it is not known whether miR-200b directly regulated intestinal fibrosis.

EMT is a process in which epithelial cells lose their phenotypic and functional characteristics while acquiring mesenchymal features. Although EMT is not a common event in adults, this process has been implicated in such instances as wound healing and fibrosis. The role of EMT in intestinal fibrosis has yet to be investigated. Intestinal fibrosis is thought to occur as a result of chronic inflammation and dysregulated wound healing (40,41). In this study, we demonstrated that EMT may promote intestinal fibrogenesis, which was probably inhibited by miR-200b (Figs. 3 and 5). In future studies, we will attempt to provide additional evidence to prove this hypothesis.

## Figures and Tables

**Table I:** Characteristics of Crohn’s disease patients.

	Age	Gender	Diagnosis	Tissue-staining	N-cadherin staining	Vimentin staining	α-SMA
Fibrosis	31	M	CD	Intestine/serum	+++	++	++
	35	M	CD	Intestine/serum	++	++	++
	47	F	CD	Intestine/serum	+	+	+
	73	F	CD	Intestine, colon/serum	++	++	++
	41	F	CD	Intestine, colon/serum	+++	+	+
	39	F	CD	Intestine/serum	+	++	++
	54	F	CD	Intestine, sigmoid/serum	+	+	+
	74	M	CD	Intestine/serum	+++	++	++
	54	M	CD	Intestine, sigmoid/serum	++	+	+
	68	F	CD	Intestine, sigmoid/serum	++	+	+
No-fibrosis	37	M	CD	Colon ascendens, intestine/serum	−	+	−
	49	M	CD	Intestine/serum	+	−	−
	17	M	CD	Intestine, colon/serum	−	−	−
	24	M	CD	Intestine/serum	−	−	−
	27	M	CD	Intestine/serum	+	−	−
	29	F	CD	Intestine/serum	−	−	−
	16	F	CD	Intestine/serum	−	−	−
	22	M	CD	Intestine/serum	−	−	−
	27	M	CD	Intestine, colon/serum	−	−	−
	24	M	CD	Intestine/serum	−	−	−

Crohn’s disease patients were divided into two subgroups, fibrosis and no fibrosis, according to the staining of α-smooth muscle actin (α-SMA) and vimentin, which are the two markers of fibrosis. (+, mild; ++, moderate; +++, strong).
